# Central Nervous System (CNS) Viral Seeding by Mature Monocytes and Potential Therapies To Reduce CNS Viral Reservoirs in the cART Era

**DOI:** 10.1128/mBio.03633-20

**Published:** 2021-03-16

**Authors:** Rosiris León-Rivera, Mike Veenstra, Maribel Donoso, Elizabeth Tell, Eliseo A. Eugenin, Susan Morgello, Joan W. Berman

**Affiliations:** a Department of Pathology, Albert Einstein College of Medicine, Bronx, New York, USA; b Department of Microbiology and Immunology, Albert Einstein College of Medicine, Bronx, New York, USA; c Department of Neuroscience, Cell Biology, and Anatomy, University of Texas Medical Branch, Galveston, Texas, USA; d Department of Neurology, Icahn School of Medicine at Mount Sinai, New York, New York, USA; e Departments of Pathology and Neuroscience, Icahn School of Medicine at Mount Sinai, New York, New York, USA; Washington University School of Medicine; Washington University School of Medicine

**Keywords:** HIV, viral reservoirs, ART, ddPCR, DNA/RNAscope

## Abstract

The human immunodeficiency virus (HIV) enters the central nervous system (CNS) within a few days after primary infection, establishing viral reservoirs that persist even with combined antiretroviral therapy (cART). We show that monocytes from people living with HIV (PLWH) on suppressive cART harboring integrated HIV, viral mRNA, and/or viral proteins preferentially transmigrate across the blood-brain barrier (BBB) to CCL2 and are significantly enriched post-transmigration, and even more highly enriched posttransmigration than T cells with similar properties. Using HIV-infected ART-treated mature monocytes cultured *in vitro*, we recapitulate these findings and demonstrate that HIV^+^ CD14^+^ CD16^+^ ART-treated monocytes also preferentially transmigrate. Cenicriviroc and anti-JAM-A and anti-ALCAM antibodies significantly and preferentially reduce/block transmigration of HIV^+^ CD14^+^ CD16^+^ ART-treated monocytes. These findings highlight the importance of monocytes in CNS HIV reservoirs and suggest targets to eliminate their formation and reseeding.

## Abstract (precis)

We characterized mechanisms of CNS viral reservoir establishment/replenishment using peripheral blood mononuclear cells (PBMC) of PLWH on cART and propose therapeutic targets to reduce/block selective entry of cells harboring HIV (HIV^+^) into the CNS. Using DNA/RNAscope, we show that CD14^+^ CD16^+^ monocytes with integrated HIV, transcriptionally active, and/or with active viral replication from PBMC of PLWH prescribed cART and virally suppressed, selectively transmigrate across a human BBB model.

## INTRODUCTION

Human immunodeficiency virus (HIV) enters and infects different tissues in the body early after infection (1), including the central nervous system (CNS), establishing viral reservoirs that persist despite combination antiretroviral therapy (cART) (2, 3). Suppressive cART inhibits active viral replication and infection of additional cells. It efficiently reduces the number of cells productively infected with HIV but does not eliminate cells harboring virus (3–9), viral reservoirs (10–13), or the production of viral proteins (14, 15). The cells with suppressed or latent virus may contribute to rebound of viral replication upon cessation of cART (16–18) and to the development of HIV-associated comorbidities in people living with HIV (PLWH), including HIV-Associated Neurocognitive Disorders (HAND), that persist in 15 to 40% of PLWH in the cART era (19, 20).

HIV enters the CNS by transmigration of HIV-infected monocytes, and perhaps T cells, across the blood-brain barrier (BBB) (21, 22). This is mediated by increased chemokines, including CCL2, in the CNS of PLWH even with cART (23, 24). Once within the CNS, monocytes may differentiate into perivascular macrophages that can constitute long-lived viral reservoirs or release infectious virus that infect additional CNS cells, including macrophages, microglia, and astrocytes, all of which can persist as reservoirs despite long-term viral suppression with cART. HIV-infected CNS cells produce host and viral factors, such as Tat and Nef, and activate other CNS cells, leading to the release of neurotoxic mediators and cytokines, resulting in low-level chronic neuroinflammation and neuronal damage (14, 15, 25–27). This chronic neuroinflammatory environment persists despite cART, mediating recruitment of additional uninfected and HIV-infected cells, contributing to replenishment of CNS viral reservoirs, and possibly enabling persistence of HAND.

A monocyte subset, characterized by surface CD14, the lipopolysaccharide coreceptor, and CD16, the FcγIII receptor, is proposed to be essential for viral reservoirs (28–33). CD14^+^ CD16^+^ monocytes are increased in the peripheral blood of PLWH despite cART (34), are permissive to HIV infection (33, 35), and preferentially transmigrate across the BBB to CCL2 (21). CD14^+^ CD16^+^ monocytes and T cells from peripheral blood mononuclear cells (PBMC) of PLWH, and from *in vitro* cultures, are heterogeneously infected with HIV. Some cells harbor virus (HIV^+^), and others are bystander cells remaining uninfected but exposed to HIV, viral proteins, and inflammatory mediators (HIV^exp^). Studies showed that increased levels of HIV DNA within PBMC, specifically within CD14^+^ CD16^+^ monocytes and not T cells, correlate with development of cognitive impairment in PLWH (36). We previously demonstrated HIV^+^ CD14^+^ CD16^+^ monocytes, matured and infected *in vitro*, preferentially transmigrate across the BBB to CCL2 compared to HIV^exp^ CD14^+^ CD16^+^ monocytes and that this selective advantage is facilitated by increased CCR2, the only known CCL2 receptor on monocytes, and the junctional proteins JAM-A and ALCAM (28).

The purpose of this study is to characterize potential mechanisms of CNS viral reservoir formation and reseeding in the cART era. Using PBMC from PLWH on stable, suppressive cART, we examine transmigration across an *in vitro* model of the human BBB, and show that there still is selective transmigration of CD14^+^ CD16^+^ monocytes. We now demonstrate that HIV^+^ PBMC transmigrate preferentially to CCL2 compared to HIV^exp^ PBMC. To characterize preferentially transmigrating PBMC further, we use DNA/RNAscope and immunofluorescence to demonstrate that CD14^+^ monocytes harboring HIV Nef DNA, HIV gag/pol mRNA, and/or HIV p24 protein are highly increased and enriched posttransmigration. In addition, using CD14^+^ CD16^+^ monocytes cultured, infected, and treated with ART *in vitro*, we recapitulate the selective transmigration of patient-obtained HIV^+^ CD14^+^ CD16^+^ monocytes across the BBB model. Cenicriviroc (CVC), a CCR2/CCR5 dual inhibitor currently in clinical trials for HAND (37), and anti-JAM-A and anti-ALCAM antibodies reduced and, in some cases, completely blocked this preferential transmigration. We propose that even with suppressive ART, ongoing entry of HIV^+^ cells into the CNS, and specifically HIV^+^ CD14^+^ CD16^+^ monocytes, is critical for maintenance of reservoirs and therefore to HAND. Thus, CCR2, JAM-A, and ALCAM may be therapeutic targets for HAND to reduce/block the selective entry of HIV^+^ monocytes into the CNS in the cART era. Our *in vitro* culture system can be used to screen therapies to eliminate or reduce transmigration of ART-treated HIV^+^ CD14^+^ CD16^+^ monocytes. This study is the first, to our knowledge, to demonstrate that monocytes from PLWH on cART with long-term suppression can carry HIV into the CNS with the potential to be reactivated/infectious, underscoring the importance of CD14^+^ CD16^+^ monocytes in HIV reservoir formation and reseeding. Our study also identifies therapeutic targets to limit the entry of HIV^+^ CD14^+^ CD16^+^ monocytes into the CNS to reduce viral dissemination and reservoir maintenance in the cART era.

## RESULTS

### Characteristics of study participants.

The demographic and clinical characteristics of study participants are summarized in Table 1. The individuals had a median age of 57 years, 9 (64.3%) were female, and 12 (85.7%) were minorities. Individuals had HIV disease for a median of 22 years at the time of study participation, and all were virally suppressed at the time of blood draw for monocyte studies (median log HIV RNA copies/ml 1.28 [1.28, 1.28]). At the time of study, 10 individuals had undetectable viral loads, 3 were detected below the limit of quantification (<20 copies/ml), and 1 had a detectable load of 32 copies/ml. The median years of documented viral suppression for 12 of the participants, as determined by review of laboratory values in the medical chart and using 50 copies/ml as a threshold for suppression, was 2.5 years (2.0, 4.0), with a range of 1 to 6 years. Two participants did not have prior virologic data available for review in the medical record.

**TABLE 1:** Characteristics of the PLWH study participants

Cohort characteristics	Median [IQR] or *n* (%), *n* = 14
Age (yr)	57.0 [51.3, 59.8]
	
Sex	
Female	9 (64.3)
Male	5 (35.7)
	
Race/ethnicity	
Black	7 (50.0)
Hispanic	5 (35.7)
White	2 (14.3)
	
Other	
Duration HIV disease (yr)	22 [14, 27]
CD4 T cell count (cells/μl)	669 [588, 801]
Nadir CD4 T cell count (cells/μl)	349 [218, 513]
Plasma viral load (log_10_ copies/ml)^*a*^	1.28 [1.28, 1.28]
Prescribed cART	14 (100.0)
Period (yr) of documented viral suppression^*b*^	2.5 [2.0, 4.0]

a Limits of quantitation for the assay = 20 copies/ml.

b Documented in the medical record by review of plasma viral load determinations; available for 12 of the 14 study participants.

### CD14^+^ CD16^+^ monocytes from PLWH on cART preferentially transmigrate across the BBB to CCL2.

**FIG 1:**
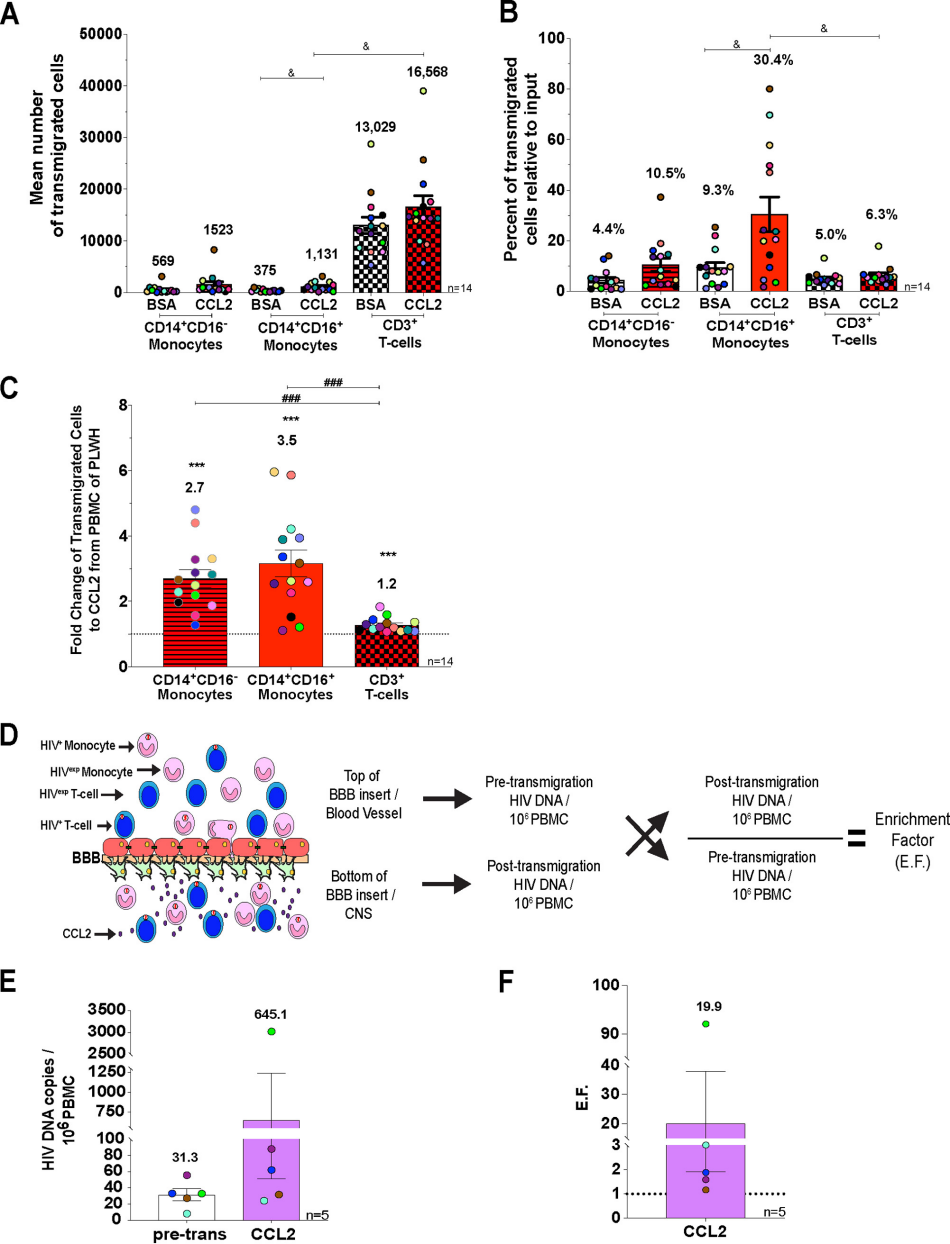
HIV^+^ PBMC from PLWH on ART appear to transmigrate preferentially across the BBB to CCL2. (A) Mean number of CD14^+^ CD16^−^ monocytes (bars with lines), CD14^+^ CD16^+^ monocytes (open bars), and CD3^+^ T cells (checkered hatching) that transmigrates across the BBB to BSA and CCL2. Significance was determined by Friedman’s test with Dunn’s multiple-comparison test. &, *P* < 0.05. (B) Percentage of CD14^+^ CD16^−^ monocytes (bars with lines), CD14^+^ CD16^+^ monocytes (open bars), and CD3^+^ T cells (checkered hatching) that transmigrated across the BBB to BSA and CCL2—relative to the number of cells for CD14^+^ CD16^−^ monocytes, CD14^+^ CD16^+^ monocytes, or CD3^+^ T cells added to the top of the BBB model—were calculated. Significance was determined by Friedman’s test with Dunn’s multiple-comparison test. &, *P* < 0.05. (C) Fold change of transmigrated CD14^+^ CD16^−^ monocytes, CD14^+^ CD16^+^ monocytes, and CD3^+^ T cells. The data are represented as the fold change relative to the baseline (BSA), set to 1. Significance was determined by Wilcoxon signed-rank test (***, *P* < 0.001) and Wilcoxon matched-pairs signed-rank test (###, *P* < 0.001). (D) Schematic representation of transmigration across the human BBB tissue culture model. The number of HIV DNA copies per 10^6^ PBMC pre- and posttransmigration were quantified by ddPCR. An enrichment factor (EF) was calculated by dividing the posttransmigration HIV DNA copies per 10^6^ PBMC by the pretransmigration HIV DNA copies. (E) Number of HIV DNA copies per 10^6^ PBMC pretransmigration (pre-trans, open bars), as well as for PBMC that transmigrated across the BBB to CCL2 (purple bars). Significance was determined by Wilcoxon signed-rank test (*P* = 0.0625). (F) The EF was calculated for the five independent individuals that were analyzed by ddPCR. Significance was determined by Wilcoxon signed-rank test (*P* = 0.0625). The data are represented as means ± the standard errors of the mean (SEM). Every donor is color-coded. Significance is compared to the baseline unless indicated otherwise.

To characterize transmigration across the BBB to CCL2 of CD14^+^ CD16^+^ monocytes and CD3^+^ T cells from PLWH on cART, we added freshly isolated PBMC from study participants to the top of our human BBB model and allowed cells to transmigrate to BSA (vehicle) or CCL2 (200 ng/ml). A portion of the cells added to the top of the barrier, referred to as pretransmigration, was analyzed using flow cytometry. After 24 h, cells that transmigrated were recovered and stained with CD14, CD16, and CD3, and monocyte and T-cell populations were quantified and characterized by flow cytometry. Monocytes and lymphocytes were identified using forward- and side-scatter areas and then analyzed for CD14 and CD16; T cells were analyzed for CD3. CCL2-mediated transmigration of CD14^+^ CD16^+^ monocytes was significantly higher than that of CD3^+^ T cells relative to their input (Fig. 1A [*P* < 0.05], Fig. 1B [*P* < 0.05], and Fig. 1C [*P* < 0.001]) from 14 independent individuals. These data indicate that CD14^+^ CD16^+^ monocytes from PLWH on cART selectively transmigrate in greater numbers across the BBB.

### HIV^+^PBMC from PLWH on cART may transmigrate selectively across the BBB in response to CCL2.

To determine whether there is selective transmigration of PBMC harboring HIV that may lead to establishment/reseeding of CNS viral reservoirs, we added PBMC to the top of the BBB model and allowed them to transmigrate to CCL2 for 24 h. We used droplet digital PCR (ddPCR), with which we can detect 1 HIV DNA copy/10^6^ PBMC to quantify HIV DNA copies per 10^6^ PBMC of the input population. After transmigration, PBMC were collected and pooled from the bottom of the wells, and 10^6^ cells analyzed by ddPCR to determine the number of HIV DNA copies per 10^6^ cells posttransmigration for 5 of the 14 individuals analyzed by flow cytometry. The remaining posttransmigration cells from 10 individuals were analyzed by a DNA/RNAscope assay, as described below. We determined that the mean number of HIV DNA copies per 10^6^ PBMC pretransmigration was 31.3 (range, 8 to 55.6; Fig. 1E), and posttransmigration it was 645.1 (range, 24 to 3,020; Fig. 1E). To determine the extent of enrichment of HIV^+^ PBMC posttransmigration, we calculated an enrichment factor (EF) for every independent individual by dividing the number of HIV DNA copies per 10^6^ PBMC posttransmigration by the HIV DNA copies per 10^6^ PBMC pretransmigration (Fig. 1D). We determined that the EF for PBMC that transmigrated was 19.9 (Fig. 1F [*P* = 0.0625]). We could only perform this analysis with cells from five individuals given the number of cells needed posttransmigration for the DNA/RNAscope assay (described below). Although we were not able to perform statistical analysis, there was a strong trend in that for each individual sample there was an increased transmigration of HIV^+^ PBMC, suggesting their preferential transmigration across the BBB.

### Monocytes that harbor HIV preferentially transmigrate across the BBB to CCL2.

**FIG 2:**
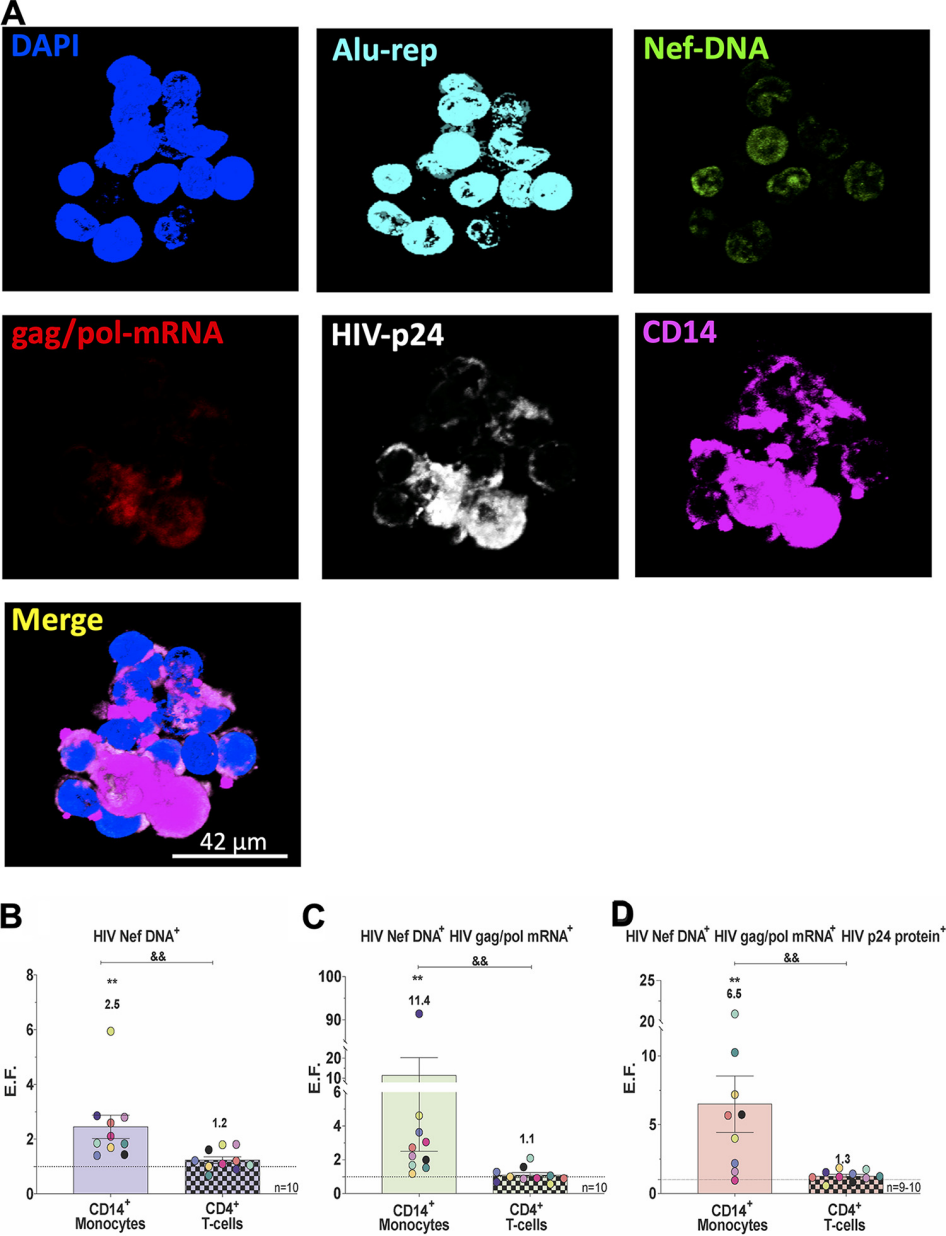
Monocytes that harbor HIV transmigrate preferentially across the BBB to CCL2. (A) Representative DNA/RNAscope images of PBMC posttransmigration stained with DAPI, DNA probes to HIV-1 Nef and nuclear Alu repeats, RNA probes to HIV-1 gag/pol, and antibodies against HIV-1 p24 and CD14. Merged scale bar, 34 μm. (B to D) The percentages of CD14^+^ monocytes and CD4^+^ T cells (Fig. S1) positive for HIV Nef DNA, HIV Nef DNA, and HIV gag/pol mRNA and for HIV Nef DNA, HIV gag/pol mRNA, and HIV p24 protein pre- and posttransmigration were calculated. A similar EF as in Fig. 1D was calculated for the percent CD14^+^ monocytes and CD4^+^ T cells positive for HIV Nef DNA (*n* = 10) (B), HIV Nef DNA and HIV gag/pol mRNA (*n* = 10) (C), and HIV Nef DNA, HIV gag/pol mRNA, and HIV p24 protein (*n* = 9 to 10) (D), as analyzed by DNA/RNAscope. Every donor is color-coded as in Fig. 1. The data are represented as means ± the SEM. Significance was determined by a Wilcoxon signed-rank test (**, *P* < 0.01) and a Wilcoxon matched-pair signed-rank test (&&, *P* < 0.01). Significance is compared to baseline unless indicated otherwise.

PBMC are heterogeneous, comprised of lymphocytes and monocytes. Monocytes and T cells can be infected with HIV, and ddPCR does not indicate which cell types are harboring virus. Thus, we optimized an *in situ* hybridization and immunofluorescence technique, the DNA/RNAscope assay, to determine which cells from PBMC harboring virus were preferentially transmigrating. Pretransmigration PBMC (10^6^) were stored until DNA/RNAscope analysis (described in Materials and Methods), and the remaining cells (4 × 10^5^/insert) were added to the top of the BBB for transmigration. After transmigration, cells were collected from the bottom of the wells and analyzed similarly. Representative images illustrating our assay are in Fig. 2A. The percentages of CD14^+^ monocytes and CD4^+^ T cells positive for HIV Nef DNA, HIV Nef DNA, and HIV gag/pol RNA and for HIV Nef DNA, HIV gag/pol RNA, and HIV p24 protein pre- and posttransmigration were quantified and analyzed by DNA/RNAscope (see Fig. S1 in the supplemental material). We calculated an EF as described above for HIV Nef DNA, HIV gag/pol mRNA, and/or HIV-p24 protein. We divided the percentage of CD14^+^ monocytes or CD4^+^ T cells posttransmigration by the percentage of CD14^+^ monocytes or CD4^+^ T cell HIV Nef DNA^+^ pretransmigration. The same EF calculations were performed for combinations of HIV Nef DNA^+^ and HIV gag/pol mRNA^+^ and of HIV Nef DNA^+^, HIV gag/pol mRNA^+^, and HIV p24 protein^+^. We found that CD14^+^ monocytes positive for HIV Nef DNA, and therefore with integrated HIV, were enriched posttransmigration (EF = 2.5, *n* = 10; Fig. 2B [*P* < 0.01]) compared to pretransmigration. In addition, we determined that CD14^+^ monocytes positive for both HIV Nef DNA and HIV gag/pol mRNA, representing cells with active viral transcription, preferentially transmigrated across the BBB to CCL2 and were highly enriched posttransmigration, with an EF of 11.4 compared to pretransmigration (*n* = 10; Fig. 2C [*P* < 0.01]). CD14^+^ monocytes with active viral production, positive for HIV Nef DNA, HIV gag/pol mRNA, and HIV p24 protein, also preferentially transmigrated across the BBB to CCL2 and were highly enriched posttransmigration with an EF of 6.5 compared to pretransmigration (*n* = 9; Fig. 2D [*P* < 0.01]). CD4^+^ T cells with similar phenotypes were not significantly enriched posttransmigration to CCL2, although they were minimally increased posttransmigration when harboring integrated HIV and/or with active viral production (Fig. 2B to D). These data demonstrate that there is highly increased preferential and selective transmigration across the BBB to CCL2 of monocytes harboring integrated virus, with active viral transcription and with active viral production. Thus, our data underscore the important role of monocytes carrying virus with the potential to be reactivated or infectious in the formation and reseeding of CNS viral reservoirs in the cART era. Elimination of these reservoirs is critical to effect a cure for HIV.

10.1128/mBio.03633-20.1 FIG S1 Increased preferential transmigration of monocytes that harbor HIV across the BBB to CCL2. The percentages of CD14^+^ monocytes (A, C, and E) and CD4^+^ T cells positive for HIV Nef DNA, HIV Nef DNA and HIV gag/pol RNA, and HIV Nef DNA, HIV gag/pol RNA, and HIV p24 protein (B, D, and F) pre- and posttransmigration were calculated as analyzed by DNA/RNAscope. Every donor is color coded as in Fig. 1. The data are represented as means ± the SEM. Significance was determined by a Wilcoxon matched-pairs signed-rank test (##, *P* < 0.001). Download FIG S1, TIF file, 1.8 MB. Copyright © 2021 León-Rivera et al. 2021 León-Rivera et al. https://creativecommons.org/licenses/by/4.0/ This content is distributed under the terms of the Creative Commons Attribution 4.0 International license.

### ART-treated HIV^+^ CD14^+^ CD16^+^ monocytes from an *in vitro* culture system preferentially transmigrate across the BBB to CCL2.

**FIG 3:**
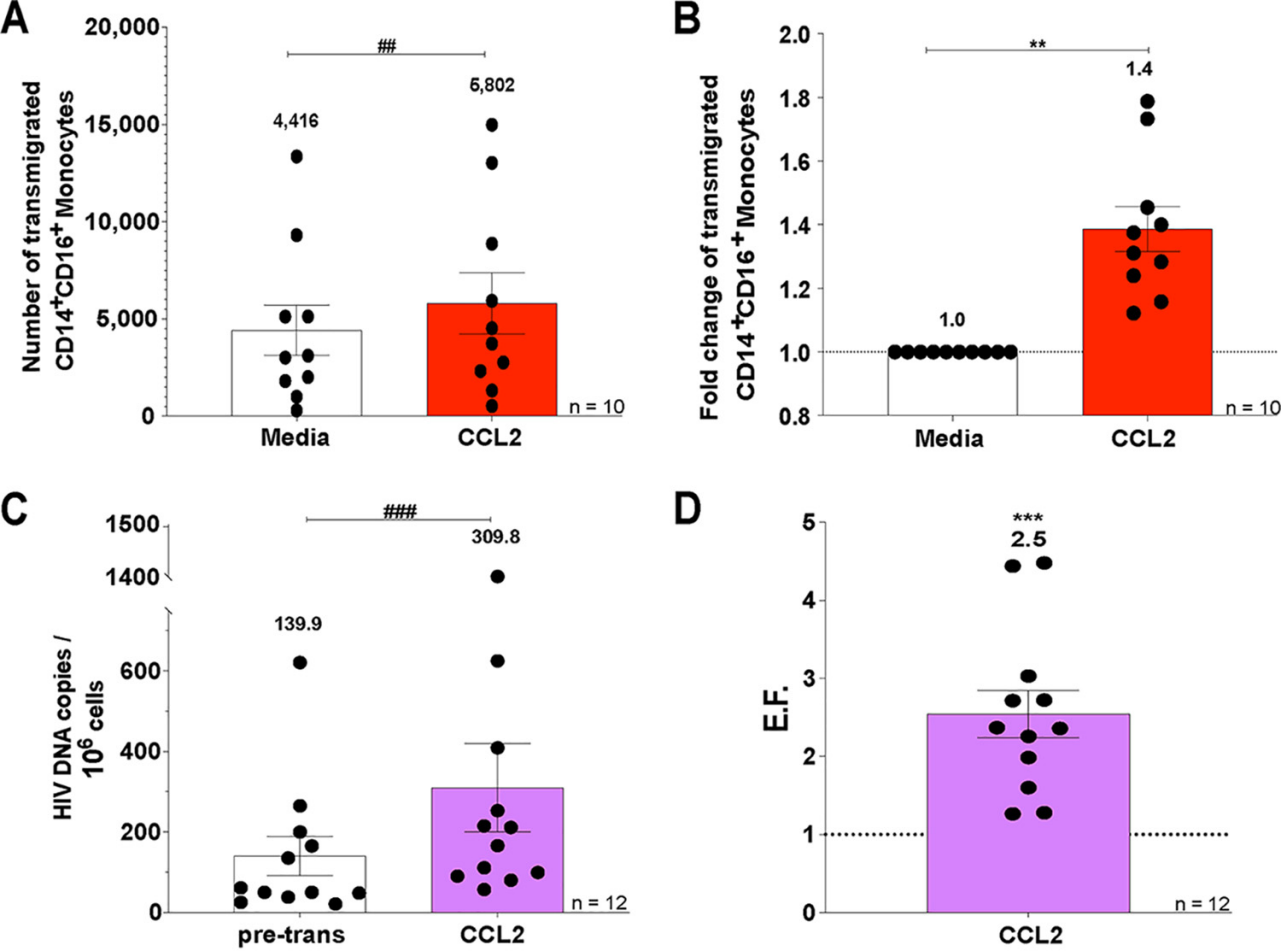
ART-treated HIV^+^ CD14^+^ CD16^+^ monocytes transmigrate preferentially across the BBB. (A and B) HIV-infected ART-treated CD14^+^ CD16^+^ monocytes from 10 independent leukopak donors were allowed to transmigrate across the BBB for 24 h in response to media (vehicle; open bar) and CCL2 (red bar). (A) The number of HIV-infected ART-treated CD14^+^ CD16^+^ monocytes that transmigrated across the BBB was determined by flow cytometry. (B) Fold change of transmigrated HIV-infected ART-treated CD14^+^ CD16^+^ monocytes relative to their baseline transmigration to media, set to 1. (C and D) The numbers of HIV DNA copies per 10^6^ HIV-infected ART-treated mature monocytes pre- and posttransmigration from 12 independent leukopak donors were quantified by ddPCR. (C) The numbers of HIV DNA copies per 10^6^ HIV-infected ART-treated mature monocytes pretransmigration (pre-trans, open bars) were determined by ddPCR, as well as for the cells that transmigrated across the BBB to CCL2 (purple bars). (D) The EF was calculated, as in Fig. 1B, for the 12 independent leukopak donors. The data are represented as means ± the SEM. Significance is compared to baseline unless indicated otherwise. Significance was determined by the Wilcoxon signed-rank test (***, *P* < 0.005) and the Wilcoxon matched-pair signed-rank test (###, *P* < 0.005).

To model transmigration of monocytes harboring virus from PLWH on cART, we used an *in vitro* culture system in which we matured CD14^+^ monocytes and obtained high numbers of primary CD14^+^ CD16^+^ monocytes as described in Materials and Methods. Briefly, PBMC were isolated from uninfected leukopaks and CD14^+^ monocytes obtained with magnetic bead nanoparticle isolation. The monocytes were cultured nonadherently for 2 days in Teflon-coated flasks with macrophage-colony-stimulating factor (M-CSF; 10 ng/ml) to obtain mature CD14^+^ CD16^+^ monocytes. The cultured CD14^+^ CD16^+^ monocytes were infected with HIV and treated 1 day later with tenofovir and emtricitabine, as described in Materials and Methods. Tenofovir and emtricitabine are a common backbone for cART and are components of the therapy of the PLWH whose PBMC were examined above. At 3 days after infection and 2 days after ART treatment, a small percentage of cells within our *in vitro* culture system was infected with HIV (HIV^+^), and the remaining cells were uninfected but exposed to viral particles, viral proteins, and inflammatory mediators (HIV^exp^). We then added this heterogeneous population (both HIV^+^ and HIV^exp^) of HIV-infected ART-treated CD14^+^ CD16^+^ monocytes (1.5 × 10^4^ cells/insert) to the top of the BBB model and allowed them to transmigrate to CCL2 for 24 h. After transmigration, the cells were collected from the bottom of the wells and analyzed by flow cytometry to determine the number of cells that transmigrated, and 10^6^ cells were analyzed by ddPCR to determine the number of HIV DNA copies per 10^6^ cells posttransmigration. The heterogeneous population of HIV-infected ART-treated CD14^+^ CD16^+^ monocytes (both HIV^+^ and HIV^exp^) transmigrated significantly more to CCL2 than to media (fold change over baseline of 1.4, *n* = 10; Fig. 3A and B [*P* < 0.01]). The mean number of HIV DNA copies per 10^6^ HIV-infected ART-treated CD14^+^ CD16^+^ monocytes from the pretransmigration population was 139.9 (*n* = 12; Fig. 3C). Posttransmigration, this level was increased to 309.8 (*n* = 12; Fig. 3C [*P* < 0.005]). We calculated an EF for every independent leukopak donor by dividing the number of HIV DNA copies per 10^6^ HIV-infected ART-treated CD14^+^ CD16^+^ monocytes posttransmigration by the HIV DNA copies per 10^6^ HIV-infected ART-treated CD14^+^ CD16^+^ monocytes pretransmigration (Fig. 1B). The EF for HIV-infected ART-treated CD14^+^ CD16^+^ monocytes that transmigrated across the BBB was 2.5 (*n* = 12; Fig. 3D [*P* < 0.005]). This indicates that ART-treated CD14^+^ CD16^+^ monocytes harboring integrated HIV (HIV^+^) preferentially transmigrate across the BBB, thus recapitulating our *ex vivo* findings by DNA/RNAscope on PBMC of PLWH with suppressive cART and underscoring the use of this culture system to test interventional strategies to eliminate CNS reservoirs.

### CCR2, JAM-A, and ALCAM are therapeutic targets to block and reduce the preferential transmigration of HIV^+^ CD14^+^ CD16^+^ monocytes treated with ART.

**FIG 4:**
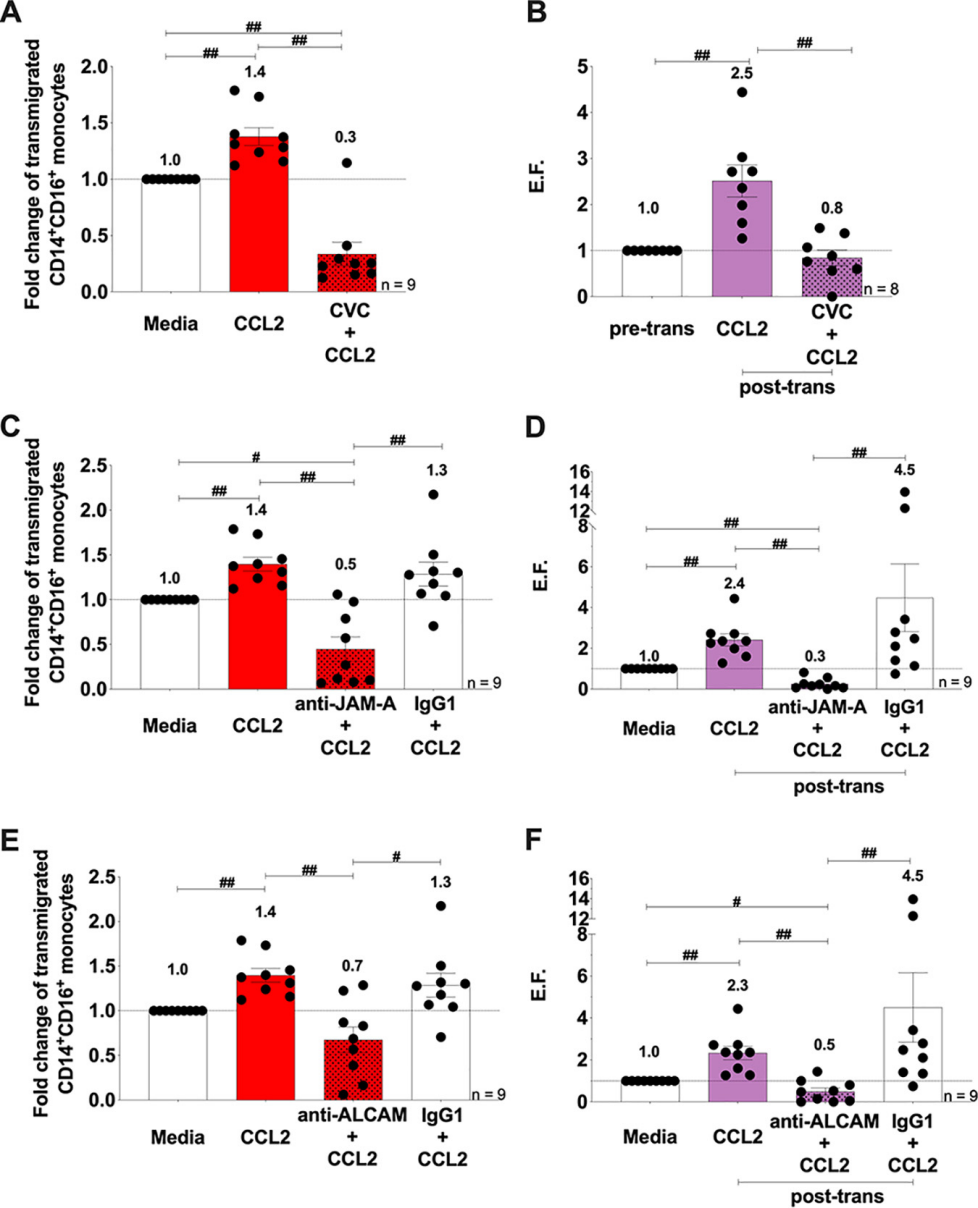
CCR2, JAM-A, and ALCAM are therapeutic targets to block and reduce the preferential transmigration of HIV^+^ CD14^+^ CD16^+^ monocytes treated with ART. (A, C, and E) Quantification by flow cytometry of the number of HIV-infected ART-treated CD14^+^ CD16^+^ monocytes that transmigrated across the BBB in the presence or absence of CVC (*n* = 9) (A), anti-JAM-A or IgG1 isotype control antibody (*n* = 9) (C), and anti-ALCAM or IgG1 isotype control antibody (*n* = 9) (E). The data are represented relative to baseline (media), set to 1. (B, D, and F) HIV DNA quantification of the number of HIV DNA copies per 10^6^ HIV-infected ART-treated mature monocytes pretransmigration (pre-trans) and posttransmigration (post-trans) by ddPCR. An EF was calculated, as in Fig. 1B, for BBB transmigration assays with CVC (*n* = 8) (B), anti-JAM-A antibody or IgG1 isotype control (*n* = 9) (D), and anti-ALCAM antibody or IgG1 isotype control (*n* = 9) (F). The data are represented as means ± the SEM. Significance was determined by the Wilcoxon’s matched-pair signed-rank test. Significance is compared to baseline unless indicated otherwise (#, *P* < 0.05; ##, *P* < 0.01).

To examine whether CCR2, JAM-A, and ALCAM are potential targets to block or reduce transmigration of HIV^+^ ART-treated CD14^+^ CD16^+^ monocytes to CCL2, we added Cenicriviroc (CVC; 100 nM), a CCR2/CCR5 dual inhibitor currently in clinical trials for HAND (37), anti-JAM-A or anti-ALCAM blocking antibodies (both at 20 μg/ml), or an IgG1 isotype-matched negative control to HIV-infected ART-treated CD14^+^ CD16^+^ monocytes and then added them to the top of the BBB model. After transmigration, cells were collected and analyzed by flow cytometry to quantify the number of cells that transmigrated and by ddPCR to determine the number of HIV DNA copies/10^6^ HIV-infected ART-treated cells posttransmigration. Using flow cytometry, we determined that CCL2 significantly increased transmigration across the BBB of the heterogenous HIV-infected ART-treated CD14^+^ CD16^+^ monocytes (both HIV^+^ and HIV^exp^) compared to media alone (fold change over media of 1.4, *n* = 9; Fig. 4A [*P* < 0.01]). CVC (fold change over media of 0.3, *n* = 9; Fig. 4A [*P* < 0.01]) and anti-JAM-A (fold change over media of 0.5, *n* = 9; Fig. 4C [*P* < 0.01]) and anti-ALCAM (fold change over media of 0.7, *n* = 9; Fig. 4E [*P* < 0.01]) antibodies significantly reduced CCL2-mediated transmigration of HIV-infected ART-treated CD14^+^ CD16^+^ monocytes. The IgG1 isotype control did not affect transmigration, indicating specificity of the blocking antibodies (fold change over media of 1.3, *n* = 9; Fig. 4C and E [*P* = 0.65]).

To assess whether our treatments blocked/reduced specifically transmigration of HIV^+^ CD14^+^ CD16^+^ monocytes treated with ART, we used our HIV DNA ddPCR assay. We found that HIV DNA copies per 10^6^ HIV-infected ART-treated CD14^+^ CD16^+^ monocytes, as indicated by the EF, were significantly increased posttransmigration (EF = 2.5, *n* = 12; Fig. 3D [*P* < 0.005]). CVC significantly reduced transmigration of HIV^+^ CD14^+^ CD16^+^ ART-treated monocytes from an EF of 2.5 to an EF of 0.8 (*n* = 8; Fig. 4B [*P* < 0.01]), and for one independent leukopak donor it completely blocked transmigration of all HIV^+^ ART-treated CD14^+^ CD16^+^ monocytes. The anti-JAM-A blocking antibody significantly reduced the transmigration of HIV^+^ ART-treated CD14^+^ CD16^+^ monocytes from all independent leukopak donors, and completely blocked, with absolutely no detectable HIV DNA posttransmigration, with cells from one independent leukopak donor. Anti-JAM-A significantly reduced transmigration of HIV^+^ CD14^+^ CD16^+^ ART-treated monocytes from an EF of 2.4 to an EF of 0.3 (*n* = 9; Fig. 4D [*P* < 0.01]). The IgG1 isotype control antibody did not significantly reduce this transmigration. Antibodies against ALCAM significantly reduced transmigration of the HIV^+^ ART-treated CD14^+^ CD16^+^ monocytes from all independent leukopak donors and completely blocked transmigration of all HIV^+^ CD14^+^ CD16^+^ ART-treated monocytes from two of the independent leukopak donors. Anti-ALCAM significantly reduced transmigration of HIV^+^ CD14^+^ CD16^+^ ART-treated monocytes from an EF of 2.3 to an EF of 0.5 (*n* = 9; Fig. 4D [*P* < 0.01]). The IgG1 isotype control antibody did not significantly reduce any transmigration (*n* = 9; Fig. 4D [*P* = 0.43] and Fig. 4F [*P* = 0.20]). These data indicate that CVC, anti-JAM-A, and anti-ALCAM blocking antibodies may significantly reduce viral entry and reentry into the CNS.

## DISCUSSION

HIV reservoirs are established early after infection, and they present a significant hurdle to an effective HIV cure. CNS viral reservoirs are established within 1 week of infection and persist even in the presence of cART (2, 3, 38–41). However, it is unclear whether persistence is static or dynamic and which cells contribute to CNS reservoir maintenance in the presence of cART. This knowledge is important to develop appropriate strategies to reduce or eliminate these reservoirs. Using PBMC from PLWH with stable viral suppression on cART, we show that PBMC harboring integrated HIV DNA appear to have a selective advantage to transmigrate across the BBB to CCL2. We are also the first to show, to our knowledge, that specifically monocytes harboring integrated virus, transcriptionally active, and/or with active viral production, and to a much lesser extent T cells, preferentially cross the barrier. Thus, selective crossing of HIV^+^ monocytes across the BBB continues in the cART era and may contribute to the persistence of HAND.

Several studies used animal models to examine mechanisms of CNS viral reservoir establishment. A study using a CD8^+^ T-cell-depleted simian immunodeficiency virus (SIV) macaque model given natalizumab, a monoclonal antibody against α4 integrin, during and after infection, showed that monocytes and not T cells contributed to CNS viral reservoir reseeding and drove neuronal injury (42). SIV-infected macaques with or without natalizumab treatment did not show CNS accumulation of CD3^+^ T cells. In addition, treatment with natalizumab 28 weeks after infection decreased monocyte accumulation within the brain, reduced the number of productively infected monocytes and macrophages within the brain, and stabilized the neuronal damage compared to untreated SIV-infected macaques (42). This study indicates an important role of monocytes in CNS reservoir reseeding, leading to neuronal damage characteristic of HAND. Another study using SIV-infected macaques virally suppressed with ART found that CD4^+^ T cells are not the only functional peripheral viral reservoir. Peripheral monocytes with integrated SIV produced replication-competent viruses when allowed to mature and differentiate into macrophages in culture (16, 43). These studies also provide evidence for spleen and lung macrophages and monocytes from PBMC as being a part of functional latent reservoirs that contribute to viral rebound following ART interruption. Specifically, within the brain these studies examined CD11b^+^ cells and found brain myeloid cells, both perivascular derived from blood monocytes and resident microglia, harboring replication-competent virus (16, 43). These authors suggest that monocyte entry into the brain, followed by the differentiation of monocytes to macrophages, may be one of the mechanisms leading to viral reservoir establishment even with cART suppression (16, 43). Our data support this hypothesis. In a study using mice reconstituted with humanized myeloid cells, myeloid-only mice (MoM), infected with HIV, there were increased human monocytes and macrophages in the brains of infected MoM compared to uninfected MoM and HIV infection in the brains of these animals (44). Given that MoM mice do not have T cells, these authors show that T cells are not necessary to traffic HIV into the brain (44). Caveats discussed in this study include that only a small percentage of monocytes harboring HIV in the periphery were detected. In addition, these authors could not distinguish between CNS viral seeding by monocyte entry or by trafficking of cell-free virus, maintaining infected resident macrophages (44). Another study using mice infected with EcoHIV, a chimeric HIV model that results in cognitive impairment and immunological characteristics similar to PLWH on successful cART, showed that EcoHIV-infected nude mice lacking T cells have increased monocytes and macrophages within the brain compared to their uninfected counterparts (45). Isolated infiltrating leukocytes from the brain tissues of these mice harbored EcoHIV, thus supporting the important role of monocytes in HIV-infection of the brain (45). Few studies have examined the role of T cells in the establishment and replenishment of viral reservoirs in the absence of monocytes, and these studies were performed without ART. One examined whether HIV infection in the brain was dependent on macrophages/monocytes using humanized T-cell-only mice (ToM). These researchers found HIV within the brains of these ToM mice, thus showing that T cells can traffic and establish viral reservoirs in the CNS in the absence of macrophages (46). A recent study using macaques infected with a neuropathogenic SIV clone, CL757, that results in progression to SIV-induced encephalitis (SIVE) in 50% of those infected, showed increased numbers of memory CD4^+^ T cells (mCD4s) and macrophages in the brains of macaques with SIVE (47). In addition, these researchers found replication-competent virus in macrophages of animals with SIVE and in mCD4s of animals with or without SIVE (47). Given that only mCD4s harbored replication-competent virus in the absence of encephalitis, these authors suggest that mCD4s may be a potential viral reservoir within the brain (47). These studies were not performed in the presence of ART and may recapitulate viral CNS infection in the pre-cART era. Overall, these studies and our findings indicate that even with successful peripheral viral suppression by cART, monocytes are important mediators of CNS infection and reseeding. Thus, we suggest that blocking CNS reseeding by HIV^+^ monocytes is crucial to the development of a successful cure.

Using PBMC from PLWH on cART, we show that monocytes harboring virus, and potentially replication-competent virus, preferentially transmigrate across the BBB to CCL2. Our DNA/RNAscope assay did not include CD16 antibody staining for detection of the CD14^+^ CD16^+^ monocytes because of limitations on the number of antibodies that can be used per sample. However, it has been shown that CD14^+^ CD16^+^ monocytes, and not CD14^+^ CD16^−^ monocytes, are a subset that is increased with HIV infection, preferentially infected by HIV, and preferentially transmigrates across our BBB model to CCL2 (12, 33, 39, 48–53). In addition, it has been demonstrated that increased HIV DNA within this mature monocyte subset correlates with the development of HAND in PLWH on cART (36). These data suggest that the monocytes harboring virus examined in our study are CD14^+^ CD16^+^.

Using an *in vitro* model to mature monocytes, infect them with HIV, and treat them with ART, we recapitulate our findings of monocytes from PBMC of PLWH on cART. We show that HIV^+^ CD14^+^ CD16^+^ ART-treated monocytes preferentially transmigrate across the BBB to CCL2. Previously, we demonstrated that surface JAM-A and ALCAM are increased on CD14^+^ CD16^+^ monocytes from PBMC of PLWH on cART and *in vitro*-cultured HIV^+^ CD14^+^ CD16^+^ monocytes and are essential for monocyte transmigration across our human BBB model (21, 28). Using our model, we now demonstrate that blocking antibodies against junctional proteins JAM-A and ALCAM significantly reduced and/or blocked the preferential transmigration of HIV^+^ CD14^+^ CD16^+^ ART-treated monocytes. Our model system enables the study and characterization of mechanisms of HIV^+^ CD14^+^ CD16^+^ monocyte selective transmigration across the BBB and can be used to test antibodies and inhibitors to block this entry into the CNS in the cART era.

Our *ex vivo* data using PBMC from PLWH on cART suggest a minimal role of T cells harboring latent virus and/or with active viral replication in viral reservoir reseeding, and our *in vitro* studies focused on targeting junctional proteins highly expressed on HIV^+^ CD14^+^ CD16^+^ monocytes. It is important to consider the impact on other cells by blocking these junctional proteins. We showed that T cells express JAM-A and that it is increased on these cells in PLWH on cART (21). However, JAM-A is expressed at lower levels on T cells than on CD14^+^ CD16^+^ monocytes and it does not play a significant role in T-cell transmigration (21). In addition, ALCAM is rarely expressed on T cells; however, it is important in CD6-mediated T-cell activation by APC (21). Thus, we hypothesize that targeting JAM-A and ALCAM will not decrease T-cell CNS surveillance and that targeting ALCAM may provide an additional benefit of decreasing T-cell activation and neuroinflammation in the cART era.

CVC is currently in clinical trials for HAND (37). When administered over a 24-week period, PLWH on cART had improved cognitive function and decreased plasma markers of monocyte immune activation (37). CVC is a CCR2/CCR5 dual inhibitory molecule, decreasing HIV infectivity of PBMC (54, 55). Our data show that CVC can decrease or block the transmigration specifically of HIV^+^ CD14^+^ CD16^+^ ART-treated monocytes. This indicates that CVC may also decrease the entry of monocytes harboring HIV into the CNS, leading to reduced reservoirs and thus decreased neuronal damage. This suggests another mechanism by which CVC may improve cognitive function.

This study demonstrates three potential targets to block reseeding of viral reservoirs in the cART era. Future studies will use combinations of anti-JAM-A, anti-ALCAM, or CVC. It will be important to characterize further differences between HIV^+^ and HIV^exp^ cells with additional state of the art technologies. Using scRNAseq (56), we showed differential gene expression between HIV^+^ and HIV^exp^ monocytes with or without cART treatment, and these may be novel targets to be examined. Future studies with combinations of treatments and/or with new targets will have to ensure that uninfected T-cell and monocyte trafficking into the CNS is not completely blocked to prevent the activation of opportunistic infections. Our study provides an *in vitro* model that can be used to test different compounds and blocking antibodies for the development of adjuvant therapies to eliminate entry of HIV^+^ monocytes and T cells into the CNS.

Our data underscore the essential role of monocytes in the cART era in viral reseeding of CNS reservoirs given their highly increased preferential and selective transmigration when harboring integrated, transcriptionally active virus and/or with active viral replication. In addition, our data suggest that targeting JAM-A, ALCAM, and CCR2/CCR5 for adjunctive therapies with preexposure prophylaxis or current cART regimens could be integral to reducing and/or preventing CNS viral reservoir replenishment and treating HAND and other comorbidities. Elimination of these reservoirs is paramount to effecting a cure for HIV infection.

## MATERIALS AND METHODS

### Study subjects, sample collection, and cell isolation.

Study participants were recruited from either outpatient infectious disease clinics at the Icahn School of Medicine at Mount Sinai (ISMMS) or ongoing studies of one of the investigators (S.M.). The present study protocols were approved by the ISMMS and Albert Einstein College of Medicine institutional review boards, and all study participants provided signed informed consent. The eligibility criteria for study included the following: adults with chronic HIV, on stable cART regimens for a minimum of 3 months with a tenofovir-emtricitabine backbone and HIV plasma RNA under 100 copies/ml; and fluency in English. Exclusion criteria included the following: HCV infection, active substance use disorder, and conditions that precluded undergoing neuroimaging procedures (i.e., metal implants, pregnancy, etc.).

Blood samples were collected in EDTA-coated tubes and processed for laboratory studies within 2 to 4 h of phlebotomy. Whole blood was layered onto Ficoll-Paque PLUS (GE Healthcare, Uppsala, Sweden) for density gradient centrifugation, and then PBMC were obtained. A PBMC sample was stained immediately for flow cytometry. In addition, one aliquot of 10^6^ pretransmigration PBMC was pelleted, washed once, fixed in 4% paraformaldehyde in 1× phosphate-buffered saline (PBS), and stored at 4°C until analysis by DNA/RNAscope assay. A second aliquot of 10^6^ pretransmigration PBMC was pelleted, washed once, and stored at −80°C until DNA isolation and quantification, and the remaining cells (400,000 cells/insert) were added to the top of BBB coculture inserts.

### Cell isolation, culture, HIV infection, and ART treatment.

PBMC were isolated by Ficoll-Paque Plus (GE Healthcare Uppsala) density gradient centrifugation from leukopaks obtained anonymously from the New York City Blood Center. Monocytes were isolated by CD14 magnetic bead-positive selection using a CD14 EasySep separation kit I (Stem Cell Technologies). Freshly isolated CD14^+^ monocytes were then cultured nonadherently for 2 days in Teflon-coated flasks at 2 × 10^6^ cells/ml in supplemented RPMI with 10 ng/ml M-CSF (Peprotech) to facilitate monocyte maturation and yield mature CD14^+^ CD16^+^ monocytes, as described previously (28, 29, 57–60). These cells were then inoculated with 1 μg/ml HIV_ADA_ at 10 × 10^6^ cells/ml in fresh media with 10 ng/ml M-CSF in Teflon-coated flasks for 8 h. After 8 h, virus was removed by centrifugation of cells, and monocytes were resuspended at 2 × 10^6^ cells/ml in fresh media with 10 ng/ml M-CSF in Teflon-coated flasks for an additional 16 h to facilitate HIV replication and infection. After an additional 16 h, ART (final concentrations of tenofovir [15 μM] and emtricitabine [15 μM]) was added directly to the media, and cells were cultured for an additional 48 h to suppress HIV replication and infection.

### Human BBB model.

The human BBB model consists of human cortical astrocytes and human brain microvascular endothelial cells (BMVEC; Applied Cell Biology Research Institute, Kirkland, WA) cocultured on opposite sides of a gelatin coated tissue culture insert with 3-μm pores (BD Falcon, Franklin Lakes, NJ), as described previously (22, 28, 29, 57, 61). Astrocytes were seeded on the bottom (CNS side) of the insert, and BMVEC were added to the top (peripheral side). The cells were grown to confluence on the inserts for 3 days, at which time astrocytic foot processes penetrate the insert and contact the BMVEC layer to create a human BBB model impermeable to albumin, 3H inulin, and with many characteristics of the human BBB (21, 22).

### BBB transmigration assay.

PBMC (4 × 10^5^) were added to the top of the BBB coculture inserts (“peripheral side”) placed in wells of a 24-well plate with CCL2 (200 ng/ml; R&D Systems) or bovine serum albumin (BSA; vehicle) added to the bottom (“CNS side”) of the inserts. Each transmigration condition was assayed with 4 replicate inserts for flow cytometry analysis and 16 to 50 replicate inserts, depending on how many PBMC were obtained, for DNA/RNAscope and ddPCR analyses. After 24 h, the transmigrated cells were collected from the bottom of the wells on the basolateral side of the inserts. For flow cytometry, collected PBMC were stained for human CD14 (1:10 dilution, clone M5E2; BD Bioscience), CD16 (2:25 dilution; clone 3G8; BD Bioscience), and CD3 (3:25 dilution; clone HIT3a; BD Bioscience) fixed with 2% paraformaldehyde in 1× PBS, and the number of transmigrated cells was quantified by flow cytometry. At least 10,000 events pretransmigration, and all events posttransmigration were acquired with the BD FACSCanto II flow cytometer. Forward- and side-scatter and CD14^+^ and CD3^+^ signals based on isotype-matched control staining and “fluorescence minus one” (FMO) controls were used to gate on monocytes and T cells, respectively. The number of each leukocyte subset that transmigrated was determined relative to the total absolute number of that subset pretransmigration, as described previously (57). Analysis was performed using FlowJo software (v10.0.8; TreeStar, Ashland, OR). For DNA/RNAscope analysis posttransmigration, transmigrated cells were collected, spun, washed once, fixed with 4% paraformaldehyde in 1× PBS, and stored at 4°C until DNA/RNAscope analysis.

### Blocking assays.

Matured HIV-infected ART-treated CD14^+^ CD16^+^ monocytes (1.5 × 10^5^) were added to the top of the BBB coculture inserts and placed in wells of a 24-well plate. Medium or CCL2 (200 ng/ml; PeproTech) was added to the wells on the bottom side of the inserts. For these experiments, we used a CCL2 that can be reconstituted with media to avoid any elevated baseline transmigration that is sometimes observed with BSA. For BBB transmigration experiments that included CVC or the blocking antibodies, mature HIV-infected ART-treated CD14^+^ CD16^+^ monocytes were added concomitantly with CVC (Allergan, 100 nM), anti-JAM-A (Santa Cruz), anti-ALCAM (Antigenix), or IgG1 antibodies (Chemocentryx) (all at 20 μg/ml) to the top of the BBB cocultures. Each transmigration condition was performed with 4 replicate inserts for flow cytometry analysis and with 40 replicate inserts for HIV DNA analysis by ddPCR. The transmigrated cells were collected from the bottom of the wells on the basolateral side of the inserts after 24 h of transmigration. For flow cytometry, HIV-infected ART-treated CD14^+^ CD16^+^ monocytes were stained for human CD14 (1:10 dilution, clone M5E2; BD Bioscience) and CD16 (2:25 dilution; clone 3G8; BD Bioscience) and fixed with 2% paraformaldehyde in 1× PBS, and the number of transmigrated cells was quantified by flow cytometry. At least 10,000 events pretransmigration and all events posttransmigration were acquired with the BD FACSCanto II flow cytometer. Analysis was performed using FlowJo software (v10.0.8; TreeStar, Ashland, OR). For HIV DNA analysis, transmigrated cells were collected, spun, counted, and stored at −80°C until DNA isolation and quantification.

### DNA/RNAscope assay.

All steps were performed in parallel on test samples and positive (ACH2 cells with only one integrated copy of HIV-1 DNA) and negative (uninfected PBMC) controls, as described previously (2, 5, 62). For negative controls scrambled probes, isotype-matched control antibodies, and DAPI (4′,6′-diamidino-2-phenylindole) were used to detect nonspecific binding of probes, antibodies, and nuclei, respectively. The following probes and antibodies were used: for HIV-1 Nef DNA detection, peptide nucleic acid (PNA) probes (NEF-Alexa 488, Alexa488-GCAGCTTCCTCATTGATGG; PNA Bio); for HIV-1 gag/pol mRNA detection, RNAscope probes RNAscope 2.5 HD detection reagent-RED (catalog no. 322360; ACD); for an internal positive hybridization control to ensure proper probe binding, Alu repeat probe (Alu-Alexa 647, Alexa647-GCCTCCCAAAGTGCTGGGATTACAG; PNA Bio); for HIV-1 p24 protein detection, anti-HIV-p24 antibody (1:500 dilution; catalog no. GTX40774; Genetex); for CD14 monocyte detection, anti-CD14 antibody clone 1H5D8 (1:500 dilution; ab181470; Abcam) with secondary antibody Alexa 647 (1:500 dilution; catalog no. A21235; Life Technologies); and for CD4 T-cell detection, anti-CD4-Alexa Fluor 680 polyclonal antibody (1:200 dilution; bs-0647R-A680; Bioss Antibodies).

Paraformaldehyde-fixed PBMC were washed in double-distilled water, and pelleted at 3,000 rpm for 2 min. For HIV-1 Nef DNA hybridization and detection, cells were placed in a humidified chamber and incubated with proteinase K (PNA Bio) diluted 1:10 in 1× Tris-buffered saline (TBS) for 10 min at room temperature. Cells were washed in dH_2_O for 3 min, followed by 95% ethanol for 20 s, and allowed to air dry for 5 min. Cells were incubated with HIV-1 Nef DNA and Alu probe diluted to 10 μM in 1× TBS and placed in a prewarmed humidity chamber at 42°C for 1 h. The temperature was then increased to 55°C for 1 h. The cells were washed in a stringent wash solution (PNA ISH detection kit; CK5201; Dako) diluted 1:60 in 1× TBS prewarmed to 55°C in a water bath for 25 min in an orbital shaker, and then washed with 1× TBS for 20 s. Afterward, HIV-1 gag/pol RNA detection was performed according to the manufacturer’s protocol for RNAscope 2.5 HD detection reagent-RED. Briefly, RNAscope probes (ACD) were added, and cells were placed in a prewarmed humidity chamber at 42°C for 30 min. The temperature was then increased to 55°C for 50 min. Next, the cells were placed in stringent wash solution (PNA ISH detection kit) diluted 1:60 in 1× TBS prewarmed to 55°C in a water bath for 15 min in an orbital shaker and then immersed in 1× TBS for 20 s. RNA was then hybridized using an RNAscope kit (ACD) with the following cycles in the incubator with washes with 1× TBS between each cycle: 30 min with RNAscope 2.5 AMP1 at 40°C, 15 min with RNAscope 2.5 AMP2 at 40°C, 30 min with RNAscope 2.5 AMP3 at 40°C, 15 min with RNAscope 2.5 AMP4 at 40°C, 30 min with RNAscope 2.5 AMP5 at room temperature, 15 min with RNAscope 2.5 AMP6 at room temperature, and 10 min with a freshly prepared mix 60:1 of RNAscope 2.5 AMP Fast RED-A and RNAscope 2.5 AMP Fast RED-B; followed by a wash with 1× TBS.

To visualize cells that were positive for CD4, CD14, and HIV p24 protein, we processed the cells as described above, first for DNA probe detection and next for RNA probe detection. The cells were then incubated in antigen retrieval solution in a water bath at 80°C for 30 min and cooled in 1× TBS, permeabilized with 0.1% Triton X-100 for 2 min, and washed three more times in 1× TBS for 5 min. Nonspecific antibody binding sites were blocked by incubating the cells in blocking solution (10% 0.5 M EDTA, 2% fish gelatin from cold water 45%, 1% albumin from bovine serum fraction V, 1% horse serum, 5% human serum, all diluted in ddH_2_O) overnight at 4°C. After blocking and permeabilization, the cells were incubated with CD4-Alexa 680-conjugated antibody (diluted 1:200) or CD14 antibody (diluted 1:500) overnight at 4°C. Cells incubated with CD14 antibody were then incubated with anti-mouse Alexa 647 as a secondary antibody for 2 h at room temperature. The cells were then washed three times in 1× TBS for 5 min to eliminate unbound antibodies. Lastly, the cells were pelleted and smeared onto glass slides, mounted with Prolong Diamond antifade mount medium containing DAPI, and stored at 4°C in the dark until microscopy and data analysis. Five to ten different images were captured for each slide using Nikon A1 confocal microscope with spectral detection, and data were analyzed using NIS-Elements AR 4.60.00 64-bit software. It is important to underscore that the computer program captured and calculated these images and numbers, respectively. In this way, we identified, quantified, and characterized low levels of HIV components (viral DNA, mRNA, and proteins) and the cell type producing them (CD14^+^ monocytes or CD4^+^ T cells). To identify HIV-infected cells, DAPI, Alu repeats, and the HIV-nef probe had to colocalize with a Pearson colocalization of 0.87. HIV mRNA and viral proteins could not be in the nucleus and had to be present in the cytoplasm. The percent CD14^+^ monocytes and CD4^+^ T cells positive for (i) HIV Nef DNA, colocalized with Alu and DAPI; (ii) HIV Nef DNA, colocalized with Alu, DAPI, and HIV gag-pol mRNA, and (iii) HIV Nef DNA, colocalized with Alu, DAPI, HIV gag-pol mRNA, and HIV p24 protein were calculated (see Fig. S1 in the supplemental material). We excluded one donor from the analysis of HIV Nef DNA, HIV gag-pol mRNA, and HIV p24 protein because it had 0% cells positive for HIV Nef DNA, HIV gag-pol mRNA, or HIV p24 protein pretransmigration, and therefore we could not calculate a reliable EF.

### ddPCR HIV DNA assay.

HIV DNA was quantified from PBMC from PLWH and from *in vitro* mature HIV-infected ART-treated CD14^+^ CD16^+^ monocytes. DNA was extracted from 10^6^ cells using the QIAamp DNA blood minikit according to the manufacturer’s protocol (Qiagen). Analysis of HIV DNA was done using ddPCR using a Bio-Rad QX-100 system as described previously (63), with a primer/probe set for HIV Gag (HIV Gag R, 5′-TCAGCCCAGAAGTAATACCCATGT-3′; HIV Gag R, 5-CACTGTGTTTAGCATGGTGTTT-3′) and HIV probe (5′-ATTATCAGAAGGAGCCACCCCACAAGA-3′; Integrated DNA Technologies).

### Statistical analysis.

Statistical analyses were performed using Prism 8 (GraphPad Software, Inc., San Diego, CA). A Wilcoxon signed-rank test was used for nonparametric measures compared to their baseline, a Wilcoxon matched-pair signed-rank test was used for paired nonparametric measures, and a Friedman test with Dunn’s multiple-comparison test was used for paired nonparametric measures to determine statistical significance (*P* < 0.05).

### Data availability.

All data are available in the text.
