# Supplementary figures and images for: Central Nervous System (CNS) Viral Seeding by Mature Monocytes and Potential Therapies To Reduce CNS Viral Reservoirs in the cART Era
Source: mBio. 2021 Mar 16;12(2):e03633-20. doi: 10.1128/mBio.03633-20 (PMC8092320; doi:10.1128/mBio.03633-20)

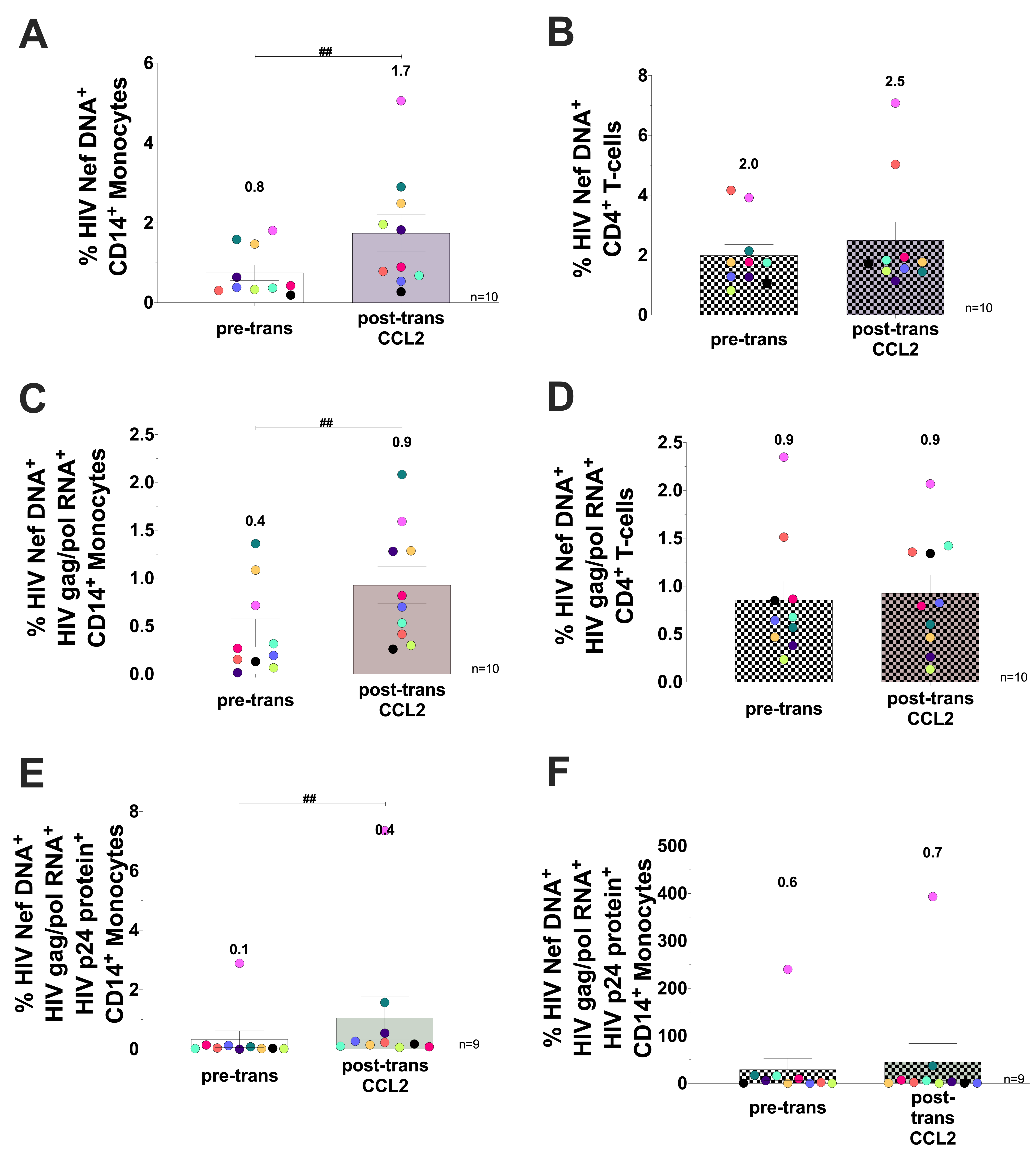

Supplement: FIG S1 [file mBio.03633-20-sf001.tif]
